# Depletion of NK6 Homeobox 3 (NKX6.3) causes gastric carcinogenesis through copy number alterations by inducing impairment of DNA replication and repair regulation

**DOI:** 10.1038/s41389-021-00365-4

**Published:** 2021-12-10

**Authors:** Jung Hwan Yoon, Jung Woo Eun, Hassan Ashktorab, Duane T. Smoot, Jeong kyu Kim, Suk Woo Nam, Won Sang Park

**Affiliations:** 1 Department of Pathology, Functional RNomics Research Center, College of Medicine, The Catholic University of Korea, Seoul, South Korea; 2 Department of Gastroenterology, Ajou University School of Medicine, Suwon, South Korea; 3 Department of Medicine, Howard University, Washington, DC USA; 4 Department of Medicine, Meharry Medical Center, Nashville, TN USA; 5 Department of Life Science, Chung-Ang University, Seoul, South Korea

**Keywords:** Gastric cancer, Genomic instability

## Abstract

Genomic stability maintenance requires correct DNA replication, chromosome segregation, and DNA repair, while defects of these processes result in tumor development or cell death. Although abnormalities in DNA replication and repair regulation are proposed as underlying causes for genomic instability, the detailed mechanism remains unclear. Here, we investigated whether NKX6.3 plays a role in the maintenance of genomic stability in gastric epithelial cells. NKX6.3 functioned as a transcription factor for *CDT1* and *RPA1*, and its depletion increased replication fork rate, and fork asymmetry. Notably, we showed that abnormal DNA replication by the depletion of NKX6.3 caused DNA damage and induced homologous recombination inhibition. Depletion of NKX6.3 also caused copy number alterations of various genes in the vast chromosomal region. Hence, our findings underscore NKX6.3 might be a crucial factor of DNA replication and repair regulation from genomic instability in gastric epithelial cells.

## Introduction

Genome stability is necessary for the integrity of human cells that are exposed continuously to the genotoxic agent. DNA repair is an important defense mechanism, maintaining genome integrity. Defective DNA repair can lead to cancer development by inducing mutations and chromosomal aberrations [1]. p53 is activated by DNA-damaged checkpoint signals and its activation coordinates various DNA damage responses to maintain genomic stability to prevent cancer development [2]. In particular, DNA double-strand breaks (DSBs), one of the most deleterious forms of DNA damage, are mostly repaired by two main mechanisms: (1) non-homologous end-joining (NHEJ), which is active in the G1 cell cycle; and (2) homologous recombination (HR), which preferentially repairs DSB in the late S/G2 phase of the cell cycle [3, 4]. Although p53 can regulate DSB repair genes, including RAD51 [5], the molecular mechanisms that underlie the formation of DNA copy number alterations (CNAs) in gastric carcinogenesis remain poorly understood.

Members of the NKX family are involved in various developmental processes, such as determining cell fate in the central nervous system, gastrointestinal tract, and pancreas [6]. In the stomach, NK6 Homeobox 3 (NKX6.3) is expressed in the epithelium of the most distal region, which eventually segregates to the lower/base region of the gastric unit [6, 7]. Previously, we reported that NKX6.3, which showed frequently lost or reduced expression in gastric cancers, acted as a gastric tumor suppressor by inhibiting cell proliferation and inducing apoptosis [8, 9]. It also protects gastric epithelial cells against the expression of the AICDA/APOBEC family, NFκB, and CBFβ gene expression, known to generate genome-wide somatic mutations and the accumulation of genetic alterations in gastric epithelial cells [10].

In the present study, we investigate subsequent effects caused by the loss of NKX6.3 on DNA replication and the DNA damage response in gastric mucosal epithelial cells to understand the molecular mechanisms underlying DNA CNAs during gastric carcinogenesis. We identify a novel role for NKX6.3 in genome maintenance, which provides new insights into its role in gastric tumorigenesis.

## Results

### Depletion of NKX6.3 aberrantly regulates DNA Replication

**Fig. 1:**
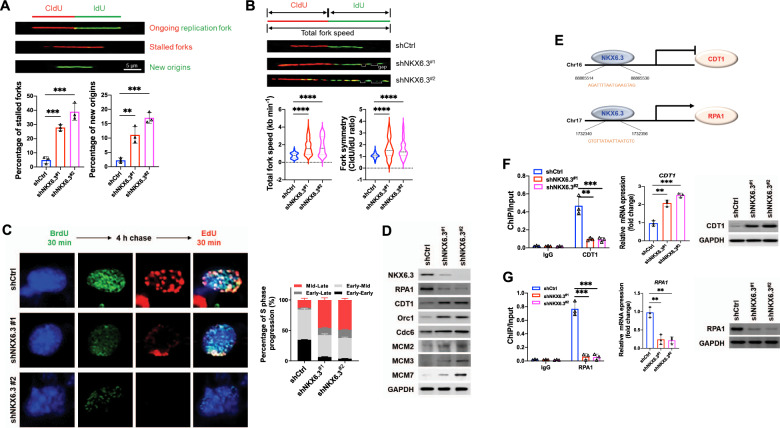
Depletion of NKX6.3 aberrantly regulates DNA replication. **A** Schematic of the general design of DNA fiber analysis. Depletion of NKX6.3 enhanced frequencies of stalled forks and newly-initiated forks (New origins; *n* = 3). **B** Representative images of DNA spreading. Lengths of IdU and CIdU tracts from DNA fibers were measured, showing the speed of fork progression and fork asymmetry in HFE-145^shCtrl^, HFE-145^shNKX6.3#1^, and HFE-145^shNKX6.3#2^ cells (*n* > 115). **C** S phase progression in HFE-145^shCtrl^, HFE-145^shNKX6.3#1^, and HFE-145^shNKX6.3#2^ cells (*n* = 3). **D** In immunoblot analysis, NKX6.3 depletion reduced expression of RPA1 but increased CDT1, Orc1, Cdc6, MCM2, MCM3, and MCM7 expression. **E** Schematic showing the CDT1 and RPA1 promoter and the NKX6.3 target regions. **F**, **G** ChIP-qPCR (left), real-time qPCR (middle), and immunoblot analysis (right) of CDT1 (**F**) and RPA1 (**G**) in HFE-145^shCtrl^, HFE-145^shNKX6.3#1^, and HFE-145^shNKX6.3#2^ cells (*n* = 3).

To test our hypothesis that NKX6.3 depletion might be associated with abnormal DNA replication, we performed DNA fiber analysis, a method of measuring DNA synthesis and replication stress (RS) levels [11]. The cells were pulse-labeled with 5-chloro-2′-deoxyuridine (CIdU; red) and 5-iododeoxyuriduine (IdU; green). Ongoing replication can be detected by the simultaneous staining of the red and green DNA strands, but the singly-labeled red or green tracts indicate stalled forks and newly-initiated forks (new origin), respectively (Fig. 1A). Compared to HFE-145^shCtrl^ cells, NKX6.3-depleted HFE-145 cells increased the frequencies of stalled forks 6- and 8-fold, and newly-initiated forks by approximately 5.5- and 8.5-fold, respectively (Fig. 1A). Moreover, the depletion of NKX6.3 increased the velocity of fork progression and fork asymmetry in the HFE-145^shNKX6.3^ cells (Fig. 1B). In an analysis of the replication program by 5-bromo-2′-deoxyuridine (BrdU)/5-ethynyl-2′-deoxyuridine (EdU) dual-labeling, markedly reduced early-to-early S phase cells but increased mid-to-late and early-to-late S phase cells were observed in the HFE-145^shNKX6.3^ cells (Fig. 1C). Furthermore, the NKX6.3 depletion increased the expression levels of CDT1, Orc1, Cdc6, MCM2, 3, and 7, while RPA1 expression was reduced (Fig. 1D). Because NKX6.3 was found to be a transcription factor for various genes [10], we asked whether NKX6.3 play a role as a transcription factor for DNA replication-related genes. ChIP analysis confirmed that the depletion of NKX6.3 increased the NKX6.3 binding activity to the promoter regions of the *CDT1* and *RPA1* genes. Then, NKX6.3 depletion increased the expression of CDT1, whereas it decreased RPA1 expression at the mRNA and protein levels (Fig. 1E–G), suggesting that NKX6.3 could act as a transcription factor for *CDT1* and *RPA1*. When we examined the effect of CDT1 overexpression and RPA1 depletion on DNA replication in the HFE-145^shCtrl^ cells, the ectopic expression of CDT1 and RPA1 deficit increased the fork progression speed (Supplementary Fig. S1A). Besides, the knockdown of CDT1 and ectopic expression of RPA1 decreased the frequencies of stalled forks and newly-initiated forks, respectively, compared to those in the HFE-145^shNKX6.3#1^ and HFE-145^shNKX6.3#2^ cells (Supplementary Fig. S1B, C). Moreover, the knockdown of CDT1 and ectopic expression of RPA1 in the NKX6.3-depleted HFE-145 cells reduced the velocity of fork progression and fork symmetry induced by NKX6.3 depletion (Supplementary Fig. S1D). These results thus suggest that NKX6.3 may modulate DNA replication via the transcriptional regulation of *CDT1* and *RPA1*.

### NKX6.3 regulates DNA damage response

**Fig. 2:**
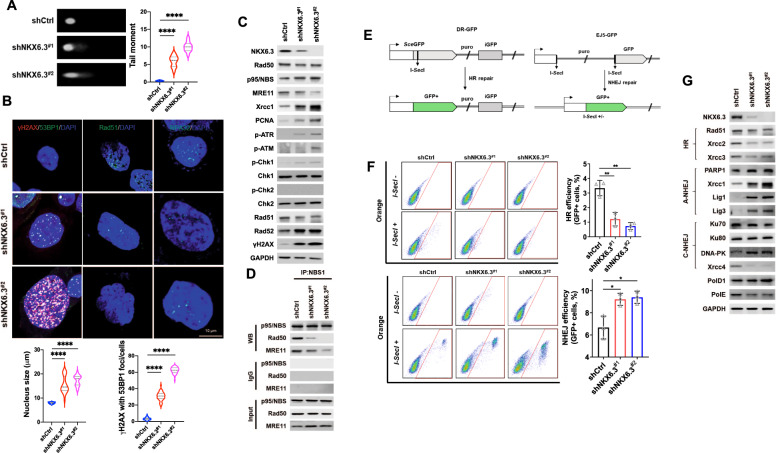
NKX6.3 regulates DNA damage response. **A** Representative comet images and the mean of tail moments in HFE-145^shCtrl^, HFE-145^shNKX6.3#1^, and HFE-145^shNKX6.3#2^ cells (*n* > 10). **B** Immunofluorescent staining for γH2AX, 53BP1, Rad51, and RPA32 in HFE-145^shCtrl^, HFE-145^shNKX6.3#1^, and HFE-145^shNKX6.3#2^ cells. Nuclear size was measured in HFE-145^shCtrl^, HFE-145^shNKX6.3#1^, and HFE-145^shNKX6.3#2^ cells (lower left; *n* > 100). Foci of cells were scored positive when at least five γH2AX foci colocalized with 53BP1 (lower right; *n* > 100). **C** Immunoblot analysis of Rad50, p95/NBS, MRE11, Xrcc1, PCNA, p-ATR, p-ATM, p-Chk1, p-Chk2, Rad51, Rad52, and γH2AX in NKX6.3-depleted cells. **D** In immunoprecipitation analysis, depletion of NKX6.3 inhibited the formation of the MRN complex. **E** DR-GFP is shown along with the HDR product that uses iGFP as the template for nascent DNA synthesis, which results in the restoration of a GFP expression cassette (left). EJ5-GFP is shown along with products of EJ between the distal DSB ends (distal-EJ) that restore the GFP expression cassette (right). **F** Representative dot-plot images of DR-GFP HFE-145^shCtrl^, HFE-145^shNKX6.3#1^, and HFE-145^shNKX6.3#2^ cells showing HR repair (upper left). Quantification of HR repair (upper right; *n* = 3). Representative dot-plot images of EJ5-GFP HFE-145^shCtrl^, HFE-145^shNKX6.3#1^, and HFE-145^shNKX6.3#2^ cells showing NHEJ repair (lower left). Quantification of NHEJ repair (lower right; *n* = 3). **H** Effects of CDT1 and RPA1 on tail moments in HFE-145^shNKX6.3#1^ and HFE-145^shNKX6.3#2^ cells (*n* > 10). **G** In immunoblot analysis, knockdown of CDT1 and ectopic expression of RPA1 reduced γH2AX expression in HFE-145^shNKX6.3#1^ and HFE-145^shNKX6.3#2^ cells.

Because of the depletion of NKX6.3-induced aberrant DNA replication, we next investigated whether NKX6.3 was involved in DNA damage repair in gastric epithelial cells. As shown in Fig. 2A, the tail moment was higher in the HFE-145^shNKX6.3#1^ and HFE-145^shNKX6.3#2^ cells than in the HFE-145^shCtrl^ cells. NKX6.3 depletion also significantly increased the nuclear binding site of DNA damage markers such as γH2AX, 53BP1, and Rad52, and nuclear size, while markedly decreased foci formation of Rad51 and RPA32 (Fig. 2B and Supplementary Fig. S2A). In addition, the depletion of NKX6.3induced DNA damage-response (DDR) gene perturbation such as an increase in the expression levels of p-ATR, p-ATM, p-Chk1, Xrcc1, PCNA, Rad52, and γH2AX; decrease in the expression of Rad50, MRE11, and Rad51 (Fig. 2C); and inhibition of the formation of the Mre11-Rad50-Nbs1 (MRN) complex (Fig. 2D). DSBs have the most deleterious effect on DNA. If it is left unrepaired, a single DSB can promote genomic instability [12]. The role of NKX6.3 in DSB repair mechanisms including HR and NHEJ as well as break-induced replication (BIR) and single-strand annealing (SSA) was tested by using the DR-GFP, EJ5-GFP, BIR-GFP, and SA-GFP reporter systems (Fig. 2E and Supplementary Fig. S2B). Depletion of NKX6.3induced HR repression and reduced the total GFP-positive population from 3.4% to 1.1% and 0.8% in the HFE-145^shNKX6.3#1^ and HFE-145^shNKX6.3#2^ cells, respectively. NHEJ was increased, along with an increase in the total GFP-positive population from 6.7% to 8.9% and 9.1% in the HFE-145^shNKX6.3#1^ and HFE-145^shNKX6.3#2^ cells, respectively (Fig. 2F). In addition, NKX6.3 depletion increased the total GFP-positive population from 3.25% to 12.74% and 16.29% for BIR and from 2.41% to 11.35% and 13.40% for SSR in the HFE-145^shNKX6.3#1^ and HFE-145^shNKX6.3#2^ cells, respectively (Supplementary Fig. S2B). In the NKX6.3-depleted HFE-145 cells, the expression levels of HR-related proteins, including Rad51, Xrcc2, and Xrcc3, were decreased, whereas the expression levels of NHEJ-related proteins, including Xrcc1, Lig 1, Lig 3, and DNA-PK, were increased (Fig. 2G).

Previously, we found over 150 target genes of NKX6.3 through ChIP cloning and sequencing [8]. The target genes were analyzed in the TFBS site (http://tfbsdb.systemsbiology.net), and CDT1 and RPA1 genes involved in DNA replication were selected for further studies. Expectedly, the ectopic expression of CDT1 and depletion of RPA1 increased the expression levels of γH2AX in the HFE-145^shCtrl^ cells (Supplementary Fig. S3A). The ectopic expression of CDT1 resulted in an increase in HR and the total GFP-positive population, whereas the depletion of RPA1 repressed HR and decreased the total GFP-positive population in the HFE-145^shCtrl^ cells (Supplementary Fig. S3A). However, the ectopic expression of CDT1 and depletion of RPA1 did not increase NHEJ in the HFE-145^shCtrl^ cells (Supplementary Fig. S3A). In the NKX6.3-depleted HFE-145 cells, the knockdown of CDT1 and ectopic expression of RPA1 significantly reduced the tail moment (Supplementary Fig. S3B). The expression of γH2AX and 53BP1 was significantly reduced, whereas the expression of Rad51 and RPA32 was markedly increased (Supplementary Fig. S3C, D). Additionally, the knockdown of CDT1 and ectopic expression of RPA1 restored the effects on HR and NHEJ by NKX6.3-depleted HFE-145 cells (Supplementary Fig. S3E). Taken together, these results suggest that NKX6.3 contribute to the repair of DSBs through the regulation of HR and NHEJ by regulating the expression of CDT1 and RPA1.

### NKX6.3 expression is associated with CDT1 and RPA1 expression in both xenograft mice and human gastric cancers

**Fig. 3:**
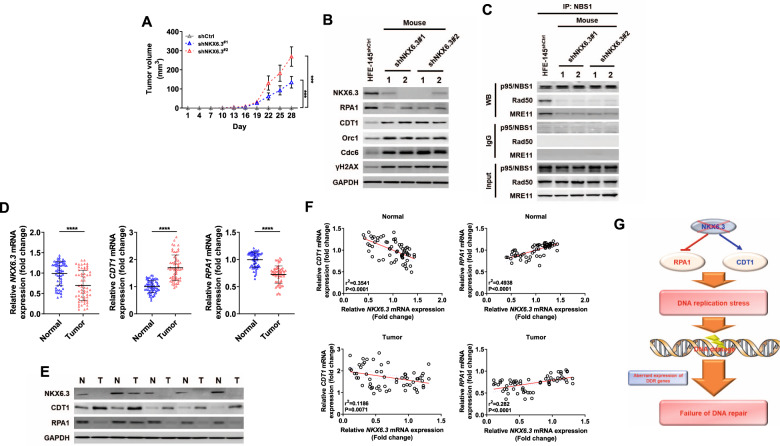
NKX6.3 expression was correlated with CDT1 and RPA1 in a xenograft model and human gastric cancers. **A** Tumor volume in mice implanted with HFE-145^shNKX6.3#1^ and HFE-145^shNKX6.3#2^ cells, whereas all mice subcutaneously injected with HFE-145^shCtrl^ cells did not develop tumors (each group; *n* = 5). **B** Immunoblot analysis of RPA1, CDT1, Orc1, Cdc6, and γH2AX in tumors derived from mice implanted with HFE-145^shNKX6.3#1^ and HFE-145^shNKX6.3#2^ cells. **C** In immunoprecipitation analysis, the failure of the MRN complex formation in tumors derived from mice implanted with HFE-145^shNKX6.3#1^ and HFE-145^shNKX6.3#2^ cells. **D**, **E** The expression levels of NKX6.3, CDT1, and RPA1 mRNA (**D**) and protein (**E**) in 60 gastric cancers. **F** NKX6.3 expression was inversely correlated with CDT1 (left) and positive correlated with RPA1 (right) in non-cancerous gastric mucosae (upper) and gastric cancers (lower; *n* = 60). **G** The schematic model hypothesized that NKX6.3 depletion leads to DNA replication stress and DNA damage by transcriptional deregulation of CDT1 and RPA1.

Consistent with our previous studies [10], mice implanted with HFE-145^shNKX6.3#1^ and HFE-145^shNKX6.3#2^ cells developed tumors (*n* = 5/5, 100%, respectively), whereas no mice subcutaneously injected with HFE-145^shCtrl^ cells developed tumors (*n* = 0/5, 0%) (Fig. 3A). In real-time RT-PCR and immunoblot analyses, the tumors derived from mice implanted with HFE-145^shNKX6.3#1^ and HFE-145^shNKX6.3#2^ cells exhibited increased CDT1, Orc1, Cdc6, and γH2AX expression, but reduced expression of RPA1 (Fig. 3B). In addition, the tumors derived from mice implanted with HFE-145^shNKX6.3#1^ and HFE-145^shNKX6.3#2^ cells did not form the Mre11-Rad50-Nbs1 (MRN) complex (Fig. 3C). In human gastric cancer tissues, the mRNA and protein expression levels of NKX6.3 and RPA1 were significantly reduced, whereas CDT1 were markedly increased. These findings were validated in the GENT2 and GEO databases (Fig. 3D, E and Supplementary Fig. S4). Next, we confirmed the association between clinicopathological parameters and mRNA expression of *NKX6.3, CDT1,* and *RPA1*. mRNA expression of *NKX6.3* was significantly decreased in TNM stages III & IV compared with TNM stages I & II (*P* = 0.024), but was not associated with gender, age, and Lauren’s classification. In addition, no association was found between clinicopathological parameters and expression of *CDT1* and *RPA1* (Supplementary Table S1). Moreover, in non-cancerous and gastric cancer tissues, the mRNA expression levels of NKX6.3 were inversely correlated with the mRNA expression levels of *CDT1*. Conversely, the expression levels of NKX6.3 were positively correlated with the *RPA1* mRNA expression levels (Fig. 3F). These results suggest that depletion of NKX6.3 in normal gastric mucosal epithelial cells causes transcriptional deregulation of CDT1 and RPA1, resulting in DNA RS and DNA damage. In addition, depletion of NKX6.3 leads to abnormal expression of various DNA damage-response genes, including the MRN complex, leading to DNA repair failure (Fig. 3G).

### Depletion of NKX6.3 leads to DNA copy number alterations (CNAs)

**Fig. 4:**
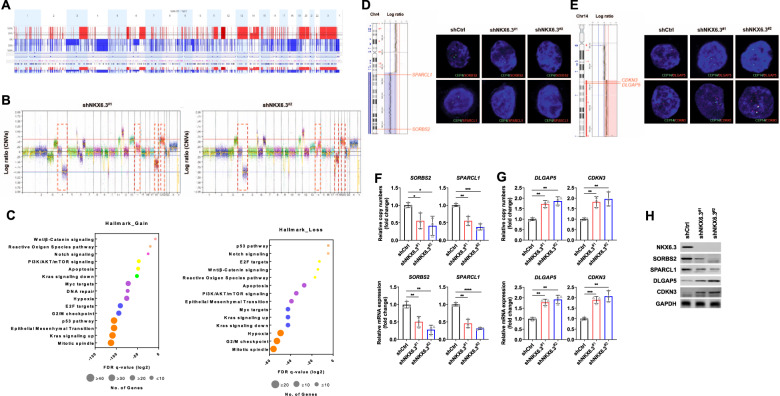
Depletion of NKX6.3 leads to DNA copy number alterations. **A** Cumulative copy-number frequencies for HFE-145^shNKX6.3#1^ and HFE-145^shNKX6.3#2^ compared to HFE-145^shCtrl^ cells. Numbers above the graph (x-axis) denote chromosomes, while frequency (y-axis) denotes the proportion of gain (red) or loss (blue) at the respective chromosomal position. **B** Whole-genome evaluation of CNAs in HFE-145^shNKX6.3#1^ and HFE-145^shNKX6.3#2^ compared to HFE-145^shCtrl^ cells is ordered sequentially by chromosomes represented by different colors. In the Log2 gene frequency graph, noticeable copy number gains and losses are displayed in red boxes. **C** Copy number gains (left) or losses (right) gene signatures were involved in 15 or 14 hallmark enrichments, respectively. **D** Loss of DNA copy number in chromosome 4, including *SORBS2* and *SPACL1*, in HFE-145^shNKX6.3#1^ and HFE-145^shNKX6.3#2^ compared to HFE-145^shCtrl^ cells (left). In FISH assay, loss of *SORBS2* and *SPACL1* gene was detected in HFE-145^shNKX6.3#1^ and HFE-145^shNKX6.3#2^ cells (right). **E** Gain of DNA copy number in chromosome 14, including *DLGAP5* and *CDKN3*, in HFE-145^shNKX6.3#1^ and HFE-145^shNKX6.3#2^ compared to HFE-145^shCtrl^ cells (left). Gain of *DLGAP5* and *CDKN3* gene was detected in HFE-145^shNKX6.3#1^ and HFE-145^shNKX6.3#2^ cells using FISH assay (right). **F**, **G** Depletion of NKX6.3 reduced DNA copy number and mRNA expression of *SORBS2* and *SPARCL1* (**F**) but increased DNA copy number and mRNA expression of *DLGAP5* and *CDKN3* (**G**) (*n* = 3). **H** Immunoblot of SORBS2, SPARCL1, DLGAP5, and CDKN3 in NKX6.3-depleted cells.

Since DSB repair abnormalities induced by DNA RS are known to cause genomic instability, including CNAs [13–15], we investigated whether depletion of NKX6.3 can induce CNAs in gastric epithelial cells. We analyzed CNAs using Nexus6.1 software (Biodiscovery) after performing whole-genome sequencing of the HFE-145^shCtrl^, HFE-145^shNKX6.3#1^, and HFE-145^shNKX6.3#2^ cells. As expected, CNAs were prominent features of the HFE-145^shNKX6.3#1^ and HFE-145^shNKX6.3#2^ cells compared to the HFE-145^shCtrl^ cells (Fig. 4A, B and Supplementary Table S2). We observed frequently gained or amplified broad chromosomal regions, including 3q, 6pq, 11q, 12p, 14q, 19q, and 20pq, and frequently lost or deleted broad chromosomal regions, including 4q, 9p, 10p, and 18pq (Fig. 4B and Supplementary Table S2). Thus, we performed hallmark enrichment analyses to determine the potential biological functions and pathways of genes with copy number gains and losses in the HFE-145^shNKX6.3#1^ and HFE-145^shNKX6.3#2^ cells using Gene Set Enrichment Analysis (GSEA) software (http://software.broadinstitute.org). Analysis of the genes with CNAs caused by NKX6.3 depletion revealed that the gained or amplified genes and the lost or deleted genes were involved in mitotic spindle formation, KRAS signaling, the G2/M checkpoint, epithelial-mesenchymal transition, PI3K/AKT/mTOR signaling, and apoptosis (Fig. 4C, and Supplementary Table S3).

When we analyzed the CNAs of 34 paired samples from The Cancer Genome Atlas Stomach Adenocarcinoma (TCGA_STAD) dataset, gained or amplified copy numbers were found in chromosomes 6, 14, and 20, and loss or deleted copy numbers were found in chromosomes 4 and 18 (Supplementary Fig. S5A). This analysis led us to focus on the CNAs in chromosomes 4, and 14. As expected, the DNA copy number of 438 genes, including *SORBS2* and *SPACL1* in chromosome 4, showed loss or deletion (Fig. 4D and Supplementary Table S4), whereas the DNA copy number of 315 genes, including *DLGAP5* and *CDKN3* in chromosome 14 showed gain or amplification in the HFE-145^shNKX6.3#1^ and HFE-145^shNKX6.3#2^ cells (Fig. 4E, F and Supplementary Table S4). Since GSEA analysis showed that genes with CNA due to NKX6.3 depletion were most closely associated with mitotic spindle formation (Fig. 4C), we verified the CNAs of genes involved in mitotic spindle formation, such as *SORBS2*, *SPARCL1*, *DLGAP5*, and *CDKN3*, using real-time PCR. The results showed that the depletion of NKX6.3 reduced the DNA copy number and mRNA expression levels of *SORBS2* and *SPARCL1* but increased the DNA copy number and mRNA expression levels of *DLGAP5* and *CDKN3* in the HFE-145^shNKX6.3#1^ and HFE-145^shNKX6.3#2^ cells (Fig. 4F, G). In Western blot analysis, the depletion of NKX6.3 decreased the expression levels of SORBS2 and SPARCL1 proteins but increased the expression levels of DLGAP5 and CDKN3 proteins in the HFE-145 cells (Fig. 4H). We further confirmed that altered DNA copy number and expression of mRNA and protein of these genes in the tumors derived from mice implanted with HFE-145^shNKX6.3#1^ and HFE-145^shNKX6.3#2^ cells (Supplementary Fig. S5B–D). Thus, we concluded that the depletion of NKX6.3 in gastric epithelial cells could induce CNAs, which subsequently affected the expression levels of various cancer-related proteins.

### NKX6.3 expression is associated with *SORBS2, SPARCL1, DLGAP5*, and *CDKN3* genes in human gastric cancers

**Fig. 5:**
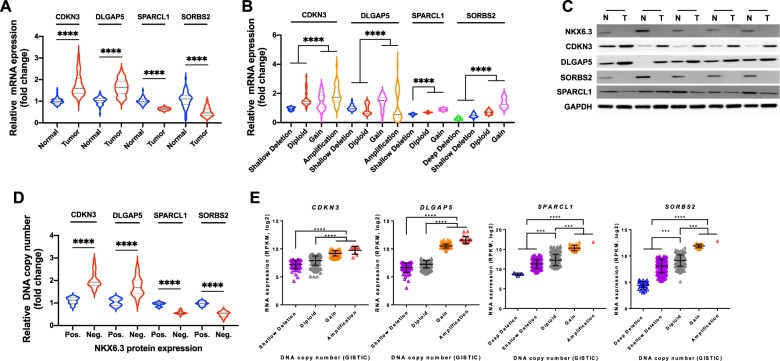
NKX6.3 expression is associated with SORBS2, SPARCL1, DLGAP5, and CDKN3 genes in human gastric cancers. **A** mRNA expression of *SORBS2*, *SPARCL1*, *DLGAP5*, and *CDKN3* in 60 human gastric cancer tissues. **B** mRNA expression of *SORBS2*, *SPARCL1*, *DLGAP5*, and *CDKN3* was positively correlated with CNAs (*n* = 60). **C** Expression of SORBS2, SPARCL1, DLGAP5, CDKN3, AurkA, and TPX2 proteins in 60 human gastric cancer tissues. **D** CNAs of *SORBS2*, *SPARCL1*, *DLGAP5*, and *CDKN3* genes was correlated with NKX6.3 protein expression (*n* = 60). **E** mRNA expression of *SORBS2*, *SPARCL1*, *DLGAP5*, and *CDKN3* was positively correlated with CNAs in the TCGA STAD data set (*n* = 380).

Since depletion of NKX6.3 in gastric epithelial cell lines induced CNAs and abnormal expression of SORBS2, SPARCL1, DLGAP5, and CDKN3, we examined NKX6.3 expression and these genes’ CNAs and expression in gastric cancer tissues. The expression levels of *CDKN3* and *DLGAP5* were markedly increased, whereas those of *SPARCL1* and *SORBS2* were decreased in 60 human gastric cancers (Fig. 5A). In addition, the mRNA expression levels of *CDKN3*, *DLGAP5*, *SPARCL1*, and *SORBS2* were positively correlated with CNAs in our gastric cancer samples (Fig. 5B). Furthermore, NKX6.3 protein expression was positively correlated with the expression of SORBS2 and SPACL1 but inversely correlated with the expression of CDKN3, and DLGAP5 proteins and CNAs (Fig. 5C, D). In addition, the mRNA expression levels of *CDKN3*, *DLGAP5*, *SPARCL1*, and *SORBS2*, were positively correlated with CNAs in the TCGA STAD data set (Fig. 5E). These results indicate that NKX6.3 depletion in normal gastric epithelial cells may activate oncogenes, such as CDKN3 and DLGAP5, and inactivate tumor suppressor genes, such as SPARCL1 and SORBS2, which subsequently lead to cancer development.

## Discussion

DNA RS, which indicates a change in the rate of replication fork progression and an increase in stalled and collapsed forks, is known to be an important mechanism causing genomic instability that can cause various diseases, including cancer [16–18]. DNA RS is an important mechanism for genomic instability [14]. It can accelerate cell growth by inactivating tumor suppressors and activating oncogenes [19]. Here, we provide evidences showing that NKX6.3 depletion could lead to DNA RS, which results in genomic instability. DNA replication is initiated by DNA binding of the origin recognition complex and the sequential binding of other replication initiator proteins [20, 21]. CDT1 and RPA1 is a key protein in the DNA replication, recombination and repair, and the activation of damage checkpoints [22–27]. Previous reports have shown that the overexpression of CDT1 and depletion of RPA1 could increase the activation of origin, induce DNA damage, stalled fork replication, defects in DNA damage repair, and promote genomic instability during tumor development [28–30]. Interestingly, we showed that NKX6.3 regulated CDT1 and RPA1 expression by acting as a transcription factor and that restoring the expression of these two proteins relieved the DNA RS caused by NKX6.3 depletion. These results demonstrate that NKX6.3 was a key factor regulating DNA replication by regulating CDT1 and RPA1 expression.

RS can lead to aberrant DNA replication fork speed and induce DNA damage [13]. DNA DSBs are a major type of DNA damage that evokes a checkpoint response and early-acting regulators, such as the MRN complex and master kinase ataxia-telangiectasia mutation (ATM), which collaborate in initiating this response cascade and repair pathway [31]. Importantly, the MRN complex can recognize DSBs and locate to the DSBs during all stages of the cell cycle [32], suggesting that the MRN complex plays an important role in checkpoint signaling from stalled replication forks. In addition, DNA DSB repair involves NHEJ or HR [33]. HR is the major error-free mechanism involved in DSB repair associated with replication forks [3]. It is active in the late S/G2 phase and involves strand invasion using the sister chromatid as a template [34]. Single-stranded DNA generated by DNA end-resection can activate ATR kinase [35], which promotes the contributions of ATR and its effector Chk1 kinase in the S and G2 phases [35]. DNA damage was shown to reduce CDT1 expression, and the overexpression of CDT1 induced re-replication, leading to DNA damage and inhibiting DNA repair [36–38]. In addition, the RPA protein is known to play an important role in DNA damage and the loss of expression and mutation of RPA1 interfere with the DNA strand exchange activity of RAD51 and lead to DSB repair failure [29, 39–41]. Notably, when RAD51, the rate-limiting factor in HR repair [42–44], becomes exhausted due to DNA damage, it leads to RAD52-dependent error-prone DNA repair pathways such as BIR and SSA [44–46]. In this study, the depletion of NKX6.3 induced the repression of HR and increased DNA damage, BIR, SSA, and NHEJ. Therefore, we hypothesized that the abnormal expression of CDT1 and RPA1 would play an important role in DNA damage and repair by NKX6.3 depletion. Expectedly, the restoration of CDT1 and RPA1 expression, which is abnormally regulated by NKX6.3 depletion, induced the recovery of DNA damage and repair induced by NKX6.3 depletion. Additionally, the expression of NKX6.3 shows a strong inverse correlation with CDT1 and a strong positive correlation with RPA1 in tumors derived from mice implanted with HFE-145^shNKX6.3#1^ and HFE-145^shNKX6.3#2^ cells and human gastric cancer tissues. All of these findings suggest that NKX6.3 acts as an important factor in regulating DNA DSB repair by modulating DNA replication and the HR system by regulating CDT1 and RPA1. Future studies are needed to determine whether DSB repair defects caused by NKX6.3 depletion could contribute to the progression of gastric cancer.

Previous studies have suggested that RS can lead to CNAs [47, 48]. CNAs occur due to errors in the repair process. For example, the HR and NHEJ of DSBs are induced by a number of factors, such as DNA RS [15]. CNAs are major genomic alterations that contribute to carcinogenesis and tumor progression caused by oncogene activation and tumor suppressor gene inactivation. Here, NKX6.3 depletion could lead to frequent gain or amplification in chromosomes 6 and 14, with loss or deletion in chromosomes 4 and 18, suggesting that NKX6.3 depletion might lead to the aberrant expression of various cancer-related proteins by inducing CNAs. The results suggest that CNAs caused by NKX6.3 depletion in normal gastric epithelial cells might lead to cancer development through activating oncogenes and inactivating tumor suppressor genes.

Herein, for the first time, we demonstrate that NKX6.3 plays a critical role in maintaining genomic stability by regulating DNA replication and DNA damage repair. NKX6.3 regulates cell-cycle progression, DNA replication, and DNA damage repair through transcriptional regulation of the novel target genes, CDT1 and RPA1. We demonstrated that depletion of NKX6.3-induced CNA, which subsequently leads to abnormal expression of cancer-related proteins, consequently promoting the development of gastric cancer. Our findings represent a NKX6.3 plays an important role in gastric carcinogenesis by controlling CNAs through regulating DNA replication and DNA repair. These findings not only partially uncovered the role of NKX6.3 in the DNA repair pathway, but also provide an invaluable basis for understanding the development of gastric cancer with DNA damage repair defects.

## Materials and methods

### Samples and ethics approval

The Chonnam National University Hwasun Hospital Biobank, a member of the Korea Biobank Network, provided a total of 60 frozen human gastric cancer tissues. The study was approved by the Institutional Review Board of The Catholic University of Korea, College of Medicine (MC15SISI0015). Written informed consent was obtained from all subjects in accordance with the Declaration of Helsinki. There was no evidence of familial cancer in any of the patients. Animal experiments were performed in mice maintained under pathogen-free conditions after approval by the Animal Experiment Ethics Committee of the Catholic University of Korea College of Medicine (CUMC-2017-0018-01).

### In vivo xenograft mouse experiment

For the in vivo xenograft assay, 5 × 10^6^ HFE-145^shCtrl^, HFE-145^shNKX6.3#1^, and HFE-145^shNKX6.3#2^ cells were mixed with 0.2 ml of phosphate-buffered saline (PBS, pH7.4) and 30% (v/v) Matrigel (BD Biosciences, San Jose, CA, USA). BALB/c-nude mice between four and five weeks old were purchased from ORIENT (Seongnam, Korea). The cells were inoculated subcutaneously into the side of the flank of the nude mice. The mice were examined twice per week for tumor formation at the injection site. After three weeks, the tumor volumes were measured every three days, and volume calculated as length × width^2^ × 0.5. After seven weeks of tumor formation, the tumors were carefully removed, photographed, and weighed. Each experimental group consisted of five mice and tumor growth was quantified by measuring the tumor sizes using calipers.

### Cell culture and transfection

An HFE-145 immortalized non-neoplastic gastric epithelial cell line was cultured at 37 °C in 5% CO_2_ in a DMEM medium with 10% heat-inactivated fetal bovine serum. The shNKX6.3 was cloned into the pGFP-C-shLenti vector (Origene, Rockville, MD, USA). The HFE-145 cells were transiently transfected with *shNKX6.3*, *siCDT1*, *siRPA1*, *Flag*-*CDT1*, *Flag*-*RPA1*, *siPTEN*, *Myc-p53 wild-type*, and *Myc-p53*^*R248W*^ in 60-mm-diameter dishes using Lipofectamine Plus transfection reagent (Invitrogen, Carlsbad, CA, USA), according to the manufacturer’s recommendations.

We generated stable NKX6.3 knockdown cells in two different target sites, HFE-145^shNKX6.3#1^ and HFE-145^shNKX6.3#2^, as well as non-targeting shRNA transfectant HFE-145shCtrl cells, as described previously [8]. The stable knockdown of NKX6.3 was confirmed in the HFE-145^shNKX6.3#1^ and HFE-145^shNKX6.3#2^ cells by Western blot analysis.

### Whole-genome sequencing and copy number data analysis

CNAs were analyzed using whole-genome sequencing (WGS), as described previously [10]. Briefly, 0.5–3 μg of DNA from the HFE-145^shCtrl^, HFE-145^shNKX6.3#1^, and HFE-145^shNKX6.3#2^ cells were used to prepare the sequencing library by shearing the DNA followed by ligating the sequencing adapters. The captured DNA was sequenced using the Illumina HiSeq X Ten platform and paired-end sequencing reads for the HFE-145^shCtrl^, HFE-145^shNKX6.3#1^, and HFE-145^shNKX6.3#2^ cells were generated. We used FastQC 0.1 software to verify the quality of the sequence reads. The paired-end library of ~500-bp inserts was sequenced with 150-bp paired-end reads. The raw sequencing data were processed with an ultrafast Isaac DNA sequence aligner designed to align the next-generation sequencing data with a low-error rate using the human genome hg19, GRCh37.

The WGS data were analyzed using Nexus Copy Number version 9.0 software (Biodiscovery Inc., El Segundo, CA, USA). The Nexus calling algorithm, SNPrank segmentation is based on the Circular Binary Segmentation model [49]. Abnormal CNVs and allele ratios were defined as the Log2 ratio values for gains and losses at 0.2 and −0.2, respectively. The thresholds for high copy number gains and homozygous deletions were set at 0.65 and −0.65, respectively.

After quantification of the genomic DNA extracted from the HFE-145^shCtrl^, HFE-145^shNKX6.3#1^, and HFE-145^shNKX6.3#2^ cells, a real-time SYBR Green quantitative polymerase chain reaction (qPCR) was performed on a Bio-Rad IQ5 real-time PCR platform (Bio-Rad Laboratories, Hercules, CA, USA). Specific primers for the analysis of the *DLGAP5*, *CDKN3*, *SORBS2*, and *SPARCL1* DNA copy numbers were designed according to GenBank’s genomic sequence. All samples were PCR amplified and normalized using oligonucleotide primers specific for constitutively expressed genes and glyceraldehyde-3-phosphate dehydrogenase (GAPDH). The primers for SYBR Green analysis were designed based on gene-specific non-homologous DNA sequences. The primer sequences are described in Supplementary Table S5.

### Fluorescence in situ hybridization

Interphase fluorescence in situ hybridization (FISH) was carried out as described [50]. Bacterial artificial chromosomes (BACs) were obtained from the BACPAC Resource Center (Oakland, CA, USA), and probes were prepared as described [51]. For the detection of *DLGAP5*, *CDKN3*, *AurakA*, *TPX2*, *SORBS2*, and *SPARCL1*, gene locus-specific probes and chromosome 4, 14, and 20-specific probes were utilized. The integrity and correct localization of all probes were confirmed by hybridization to the HFE-145^shCtrl^, HFE-145^shNKX6.3#1^, and HFE-145^shNKX6.3#2^ cells. The slides were examined using an ImagingZ1 microscope (Carl Zeiss, Oberkochen, Germany).

### DNA fiber assay

The cells were pulse-labeled with 25 μM CIdU (Sigma-Aldrich, St. Louis, MO, USA) for 30 min, then gently washed with fresh pre-warmed medium and exposed to hydroxyurea (2 mM) or untreated media, and a second pulse of 250 μM IdU (Millipore-Sigma) for 30 min. The DNA fibers were spread as described [52] before the standard detection of the CIdU and IdU tracts. For all experimental conditions, three slides were stretched and stained for each condition. CIdU was detected by a rat anti-BrdU antibody (Abcam, ab6326) and IdU by a mouse anti-BrdU antibody (Abcam, ab8152). DyLight 550 anti-rat (ThermoFisher Scientist, Waltham, MA, USA) and Alexa Fluor 488 anti-mouse (Invitrogen) secondary antibodies were used. The fibers were imaged using a Carl Zeiss LSM800 w/Airyscan confocal microscope and analyzed using ImageJ software. Statistical analyses were conducted using Prism software.

### Replication program

For double labeling, cells grown on coverslips were pulse-labeled with 10 μM BrdU for 30 min and washed and incubated in a fresh medium for 4 h. After the chase, the cells were incubated with 10 μM EdU for 30 min. The cells were then fixed in methanol. DNA was denatured in 2 M HCl for 30 min and BrdU was detected using a mouse anti-BrdU antibody and the appropriate secondary antibody. EdU was detected using the Click-iT EdU Alexa Fluor 568 Imaging Assay Kit (Life Technology, Carlsbad, CA, USA) according to the manufacturer’s protocol. High content Carl Zeiss LSM800 w/Airyscan microscopy was used for S phase analysis. The replication and S phase patterns were clustered into early, mid or late groups in the scatter plots of BrdU and EdU versus DNA content.

### Comet assay

The cells were collected and resuspended in PBS at a density of 5,000 per μl. Samples of 10 μl were mixed with 100 μl of 37 °C low melting point agarose in PBS and spotted on the slides pre-coated with normal melting point agarose in ddH_2_O. Slides were covered with coverslips and kept flat for 10 min at 4 °C. The coverslips were gently removed and the slides were immersed in cold alkaline electrophoresis buffer at 4 °C for 20 min and electrophoresis was performed at 4 °C for 25 min. The slides were then held for 10 min at 4 °C, and washed with cold PBS and cold ddH_2_O. Next, the slides were dehydrated by continuous 5 min washes in a cold graded-ethanol series (70%, 90%, and 100%) at 4 °C, air-dried, and stored at room temperature. The following day, the slides were rehydrated in ddH_2_O and stained with SybrGold for 5 min at room temperature. After washing with PBS, the slides were mounted with Vectashield mounting reagent. The images were acquired using a Carl Zeiss LSM800 w/Airyscan microscope.

### Double-strand break repair analysis

HFE-145^shCtrl^, HFE-145^shNKX6.3#1^, and HFE-145^shNKX6.3#2^ cells were established by transfecting with pDR-GFP, SA-GFP, BIR-GFP, and EJ5-GFP using Lipofectamine 2000 and selection using 1.5 μg/ml puromycin. The puromycin-resistant cells were cloned by limiting dilution in 96-well plates. Single colonies were plated in 12-well plates and transfected with cBAS to express I-SceI endonuclease. The expression of I-SceI produces site-specific double-strand breaks within one of the mutated GFP genes, which results in a functional GFP gene when recovered by gene conversion. The homologous recombination results can be measured as the percentage of GFP-positive HFE-145^shCtrl^, HFE-145^shNKX6.3#1^, or HFE-145^shNKX6.3#2^ cells. Briefly, HFE-145^shCtrl^, HFE-145^shNKX6.3#1^, and HFE-145^shNKX6.3#2^-DR-GFP, HFE-145^shCtrl^, HFE-145^shNKX6.3#1^, and HFE-145^shNKX6.3#2^-SA-GFP, HFE-145^shCtrl^, HFE-145^shNKX6.3#1^, and HFE-145^shNKX6.3#2^-BIR-GFP, and HFE-145^shCtrl^, HFE-145^shNKX6.3#1^, and HFE-145^shNKX6.3#2^-EJ5-GFP cells were sequentially transfected with the pCBASce vector. Two days later, the GFP-positive cells were analyzed by fluorescence-activated cell sorting (FACS) analysis and fluorescence-microplate reader (Promega). The Relative Fluorescence Unit was calculated by dividing the GFP RFU value by the DAPI RFU value.

### Co-immunoprecipitation (Co-IP)

Co-immunoprecipitations were performed as follows. Cells in one nearly confluent 10 cm dish were harvested in 1 ml of protein lysis buffer as described above. Subsequently, BSA-blocked protein G beads (Fastflow; Millipore-Sigma) were added and incubated at 4 °C for 30 min to preclear the lysates. One percent of the total dose was separated and rotated for 1 h at 4 °C in the presence of 2 μg of anti-p53, anti-PI3K p85α, anti-Myc-taq, and BSA-blocking protein G beads to allow immunoprecipitation. After washing the beads with lysis buffer, the proteins were eluted using Laemmli loading buffer and immunoblots were performed as described above.

### Statistical analyses

The MTT, colony formation assays, FACS, and western assays were performed at least twice, and the means ± SD are displayed in the bar graphs. One- or two-way ANOVA was conducted to assess the differences between the means. GraphPad Prism version 7.00 for Windows (GraphPad Software, La Jolla, CA, USA) was used for the statistical analyses. All other data were analyzed using the student’s *t*-test. Statistical significance is indicated as asterisks in figures: *P ≤ 0.05, **P ≤ 0.01, ***P ≤ 0.001, and ****P ≤ 0.0001.

## Supplementary information

Supplementary information

Figure S1

Figure S2

Figure S3

Figure S4

Figure S5

Table S1

Table S2

Table S3

Table S4

Table S5

Table S6
