# Supplementary material for: Depletion of NK6 Homeobox 3 (NKX6.3) causes gastric carcinogenesis through copy number alterations by inducing impairment of DNA replication and repair regulation
Source: Oncogenesis. 2021 Dec 10;10(12):85. doi: 10.1038/s41389-021-00365-4 (PMC8664813; doi:10.1038/s41389-021-00365-4)
Supplement: Supplementary file 1 — Supplementary information [file 41389_2021_365_MOESM1_ESM.docx]

**Supplementary Materials and methods**

**Fluorescence *In Situ* Hybridization**

Interphase fluorescence in situ hybridization (FISH) was carried out as described^1^. Bacterial artificial chromosomes (BACs) were obtained from the BACPAC Resource Center (Oakland, CA, USA), and probes were prepared as described^2^. For the detection of *DLGAP5*, *CDKN3*, *SORBS2*, and *SPARCL1*, gene locus-specific probes and chromosome 4 and 14-specific probes were utilized. The integrity and correct localization of all probes were confirmed by hybridization to the HFE-145^shCtrl^, HFE-145^shNKX6.3#1^, and HFE-145^shNKX6.3#2^ cells. The slides were examined using an ImagingZ1 microscope (Carl Zeiss, Oberkochen, Germany).

**Chromatin immunoprecipitation (ChIP)**

For assessing the NKX6.3 binding activity in the promoter region of *CDT1* and *RPA1*, ChIP assays were performed using the Thermo Scientific Pierce Agarose ChIP kit (Thermo Scientific Pierce, Rockford, IL, USA), as described previously^3^. DNA amplification was performed by PCR using primers for the *CDT1* and *RPA1* promoter described in Table S5. Amplification products were separated on a 2% agarose gel.

**Real-time RT-qPCR**

RNA was isolated from the cells using the RNeasy kit (Qiagen, Valencia, CA, USA) and reverse transcribed to cDNA (Life Technologies, Carlsbad, CA, USA). qPCR was performed for quantification using standard procedures on a Bio-Rad IQ5 real-time PCR platform. The mean mRNA expression value in non-neoplastic gastric tissues was used as a control. The mRNA expression change in each case was further normalized to the mean value in non-neoplastic gastric tissues. Decreased or increased mRNA expression was indicated by less than 0.5-fold or greater than 1.5-fold changes, respectively. All primers are described in Table S5.

**Immunoblotting and Immunofluorescence**

The effects of NKX6.3 on the expression of SORBS2, SPACL1, DLGAP5, CDKN3, DNA damage related genes, cell cycle-regulators, and DNA replication regulators were determined in the HFE-145^shCtrl^, HFE-145^shNKX6.3#1^, HFE-145^shNKX6.3#2^ cells, and xenograft tumor tissues by immunoblots, immunofluorescence (IF), and confocal microscopy as previously described^4^. Briefly, the cells, and mice and human gastric tissues were washed twice with cold PBS and lysed in RIPA buffer containing 50 mM Tris pH 7.4, 150 mM NaCl, 1% NP-40, 0.5% sodium deoxycholate, 0.1% sodium dodecyl sulfate (SDS), 1 mM phenylmethyl sulfonyl fluoride, and a complete protease and phosphatase inhibitor cocktail (Roche, Indianapolis, IN, USA) for 5 min and collected by scraping. The lysates were cleared by centrifuging at 12,000 rpm for 10 min. Protein was quantified by the BCA Assay and the sample proteins separated by SDS-polyacrylamide gel electrophoresis (PAGE). Next, the proteins were transferred from the gels to polyvinylidene fluoride (PVDF) membranes (Bio-Rad, Hercules, CA, USA). The membranes were blocked in 5% bovine serum albumin (BSA)-Tris-buffered saline Tween 20 (TBST) for 30 min at room temperature, and then incubated them overnight with the indicated primary antibodies at 4°C. After that, the membranes were washed three times, incubated with peroxidase-conjugated secondary antibodies (Sigma, St. Louis, MD, USA), and the immunoreactions were detected by enhanced chemiluminescence (Millipore-Sigma, Billerica, MA, USA) on a LAS-4000.

The effect of NKX6.3 on γH2AX and 53BP1 protein expression in the HFE-145^shCtrl^, HFE-145^shNKX6.3#1^, HFE-145^shNKX6.3#2^ cells was analyzed by immunofluorescence and confocal microscopy as previously described^4^. In brief, HFE-145^shCtrl^, HFE-145^shNKX6.3#1^, HFE-145^shNKX6.3#2^ cells were incubated with rabbit anti-γH2AX (Millipore-Sigma, Billerica, MA, USA), mouse anti-53BP1, rabbit anti-Rad51, mouse anti-Rad52 and rabbit anti-RPA32 (Cell Signaling Technology, Danvers, MA, USA) antibodies in PBS containing 0.5% Triton X-100 overnight. The tissue slides were rinsed with PBS and incubated with Alexa-488 conjugated goat anti-rabbit IgG (Invitrogen) or Texas-Red conjugated goat anti-mouse IgG (Invitrogen) for 1 h at room temperature. The cell nuclei were counterstained by incubating the cells with DAPI (4',6-diamidino–2'-phenylindole; dilution 1:1000; Roche) for 10 min. The slides were viewed with a Carl Zeiss LSM800 w/Airyscan confocal microscope (Carl Zeiss Co., Ltd., Germany). The images were converted to a TIFF format, and the contrast levels were adjusted using Adobe Photoshop v. 7.0 (Adobe Systems, San Jose, CA, USA). All antibodies are described in Table S6.

**Supplementary Reference**

1. Tomlins, S.A., et al. Distinct classes of chromosomal rearrangements create oncogenic ETS gene fusions in prostate cancer. *Nature*. **448**, 595-599 (2007).

2. Li, L., Chaudhuri, A., Chant, J. & Tang, Z. PADGE: analysis of heterogeneous patterns of differential gene expression. *Physiol. Genomics.* **32**, 154-159 (2007).

3. Yoon, J.H., et al. NKX6.3 controls gastric differentiation and tumorigenesis. *Oncotarget*. **6**, 28425-28439 (2015).

4. Yoon, J.H., et al. Multiple genetic mutations caused by NKX6.3 depletion contribute to gastric tumorigenesis. *Sci. Rep*. **8**, 17609 (2018).

**Supplementary Figure Legends**

**Figure S1. Effect of CDT1 and RPA1 regulated by NKX6.3 on fork speed. A** Immunoblot analysis showed expression of CDT1 and RPA1 in Flag-*CDT1* and *siRPA1* transfected HFE-145^Ctrl^ cells. Effects of ectopic CDT1 expression and RPA1 silencing on fork speed in HFE-145^Ctrl^ cells (n > 100). **B** The expression of CDT1 and RPA1 in HFE-145^shNKX6.3#1^ and HFE-145^shNKX6.3#2^ cells with siCDT1 and Flag-RPA1. **C** Effects of CDT1 and RPA1 on stalled forks and newly initiated forks in HFE-145^shNKX6.3#1^ and HFE-145^shNKX6.3#2^ cells (n = 3). **D** Effects of CDT1 and RPA1 on speed of fork progression and fork asymmetry in HFE-145^shNKX6.3#1^ and HFE-145^shNKX6.3#2^ cells (n = 137).

**Figure S2. Effects of NKX6.3 on BIR and SSA repair. A** Immunofluorescence analysis of Rad52 foci formation by NKX6.3 depletion. **B** Increased BIR activity in HFE-145^shNKX6.3#1^, and HFE-145^shNKX6.3#2^ cells. Measurement of fluorescence in HFE-145^shCtrl^, HFE-145^shNKX6.3#1^, and HFE-145^shNKX6.3#2^ cells stably expressing a BIR-GFP report vector and following I-SceI-induced DSBs shows increased BIR activity in NKX6.3-depleted cells (n = 3; left). In addition, increased SSA activity in HFE-145^shCtrl^, HFE-145^shNKX6.3#1^, and HFE-145^shNKX6.3#2^ cells. Measurement of fluorescence in HFE-145^shCtrl^, HFE-145^shNKX6.3#1^, and HFE-145^shNKX6.3#2^ cells stably expressing a SA-GFP report vector and following I-SceI-induced DSBs shows increased SSA activity in NKX6.3-depleted cells (n = 3; right).

**Figure S3. Effect of CDT1 and RPA1 on DNA repair in normal gastric epithelial cells. A** Immunoblot analysis of γH2AX expression by ectopic CDT1 expression and RPA1 silencing in HFE-145^Ctrl^ cells. Quantification of HR and NHEJ repair (n = 3). **B** In immunoblot analysis, knockdown of CDT1 and ectopic expression of RPA1 reduced γH2AX expression in HFE-145^shNKX6.3#1^ and HFE-145^shNKX6.3#2^ cells. Effects of CDT1 and RPA1 on tail moments in HFE-145^shNKX6.3#1^ and HFE-145^shNKX6.3#2^ cells (n > 10). **C** Percentage of cells with more than five γH2AX, 53BP1, Rad51, and RPA32 foci in HFE-145^shCtrl^, HFE-145^shNKX6.3#1^, and HFE-145^shNKX6.3#2^ cells (n = 3). **D** Percentage of cells with more than five γH2AX, 53BP1, Rad51, and RPA32 foci in HFE-145^shNKX6.3#1^ and HFE-145^shNKX6.3#2^ cells with siCDT1 and Flag-RPA1 (n = 3). **E** Effects of CDT1 and RPA1 on HR and NHEJ repair in HFE-145^shNKX6.3#1^ and HFE-145^shNKX6.3#2^ cells (n = 3).

**Figure S4. Expression of NKX6.3, CDT1 and RPA1 in large cohort human gastric cancers.** Validation of NKX6.3, CDT1, and RPA1 expression in human gastric cancers using GENT2 and GEO database.

**Figure S5. The CNAs of 34 paired samples in the TCGA_STAD dataset, and CNAs and mRNA expression of *SORBS2*, *SPARCL1*, *DLGAP5*, and *CDKN3*. A** Chromosomes 6, 14, and 20 showed a higher percentage of gained or amplified copy numbers, while chromosomes 4 and 18 showed a higher percentage of lost or deleted copy numbers. **B-D** DNA copy number (**B**), mRNA (**C**) and protein (**D**) expression of *SORBS2*, *SPARCL1*, *CDKN3*, and *DLGAP5* genes in tumors derived from mice implanted with HFE-145^shNKX6.3#1^ and HFE-145^shNKX6.3#2^ cells (n = 5).
