# Supplementary figures and images for: Depletion of NK6 Homeobox 3 (NKX6.3) causes gastric carcinogenesis through copy number alterations by inducing impairment of DNA replication and repair regulation
Source: Oncogenesis. 2021 Dec 10;10(12):85. doi: 10.1038/s41389-021-00365-4 (PMC8664813; doi:10.1038/s41389-021-00365-4)

**A**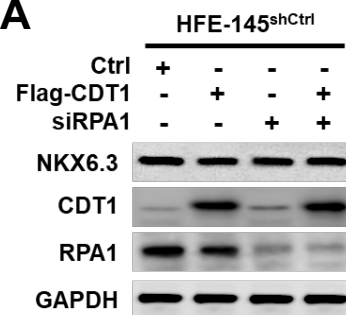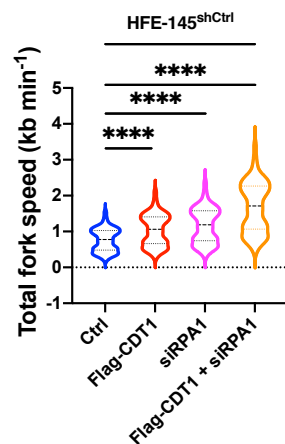**B**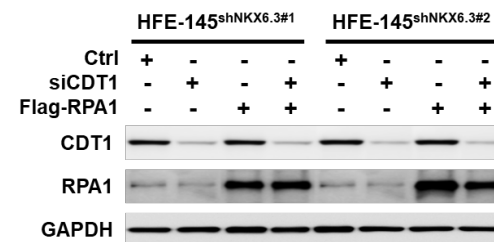**C**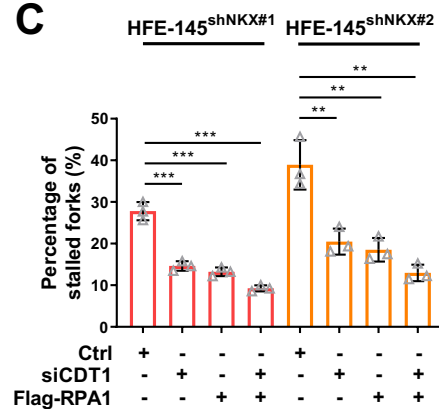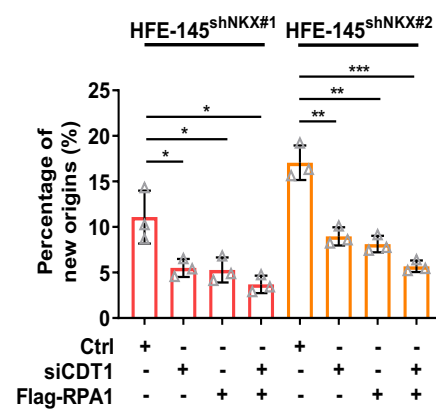**D**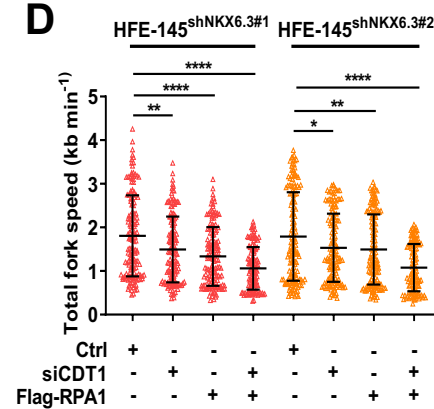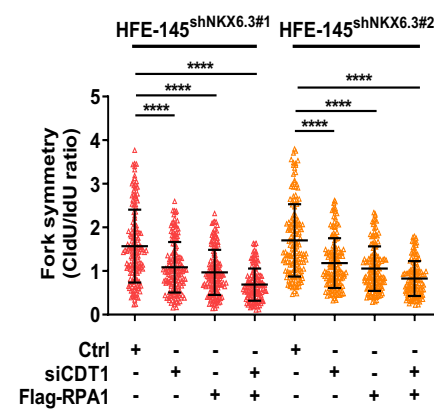

Supplement: Supplementary file 2 — Figure S1 [file 41389_2021_365_MOESM2_ESM.pdf]

**A**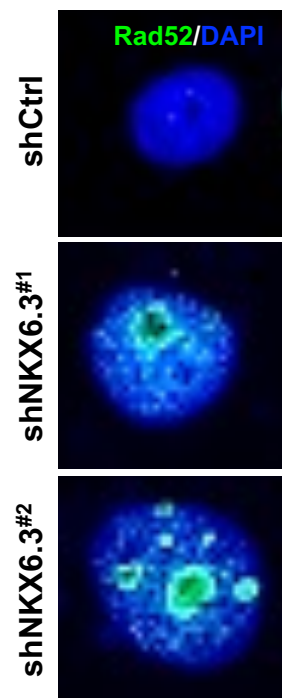**B**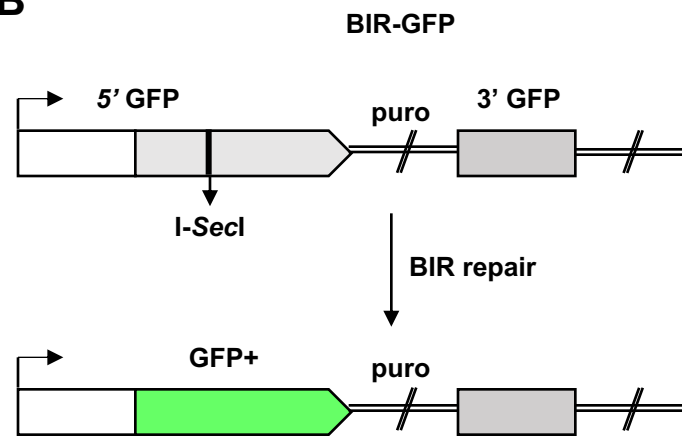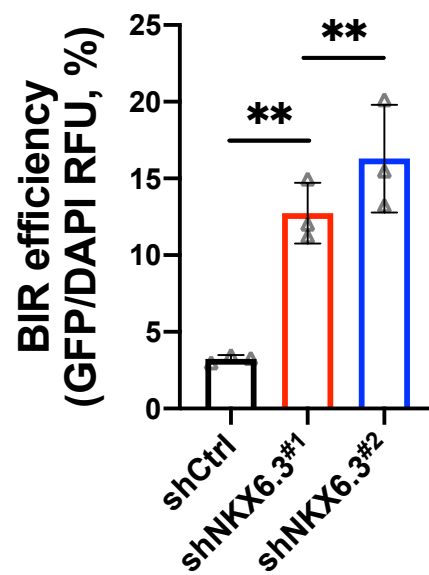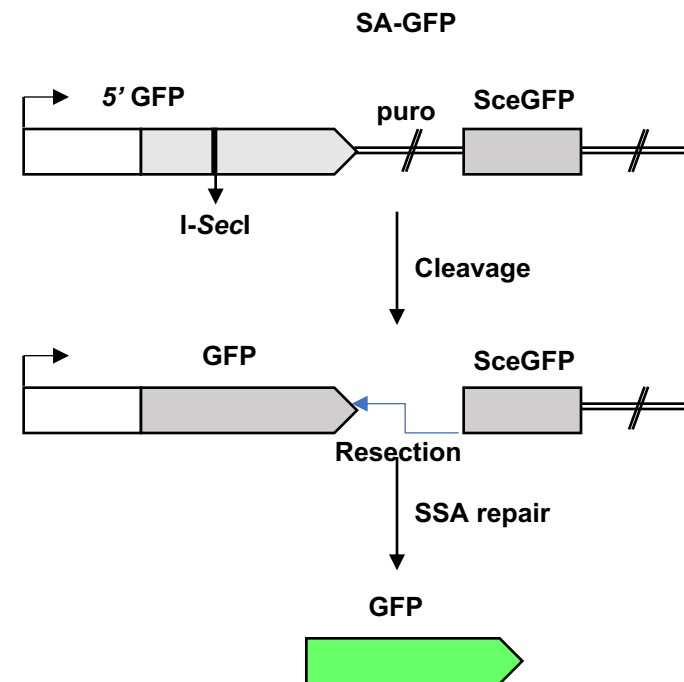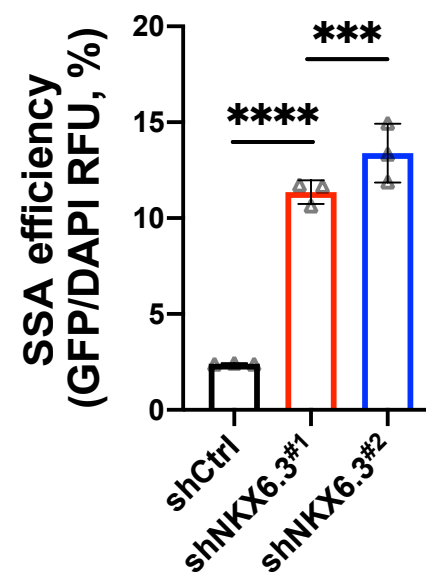

Supplement: Supplementary file 3 — Figure S2 [file 41389_2021_365_MOESM3_ESM.pdf]

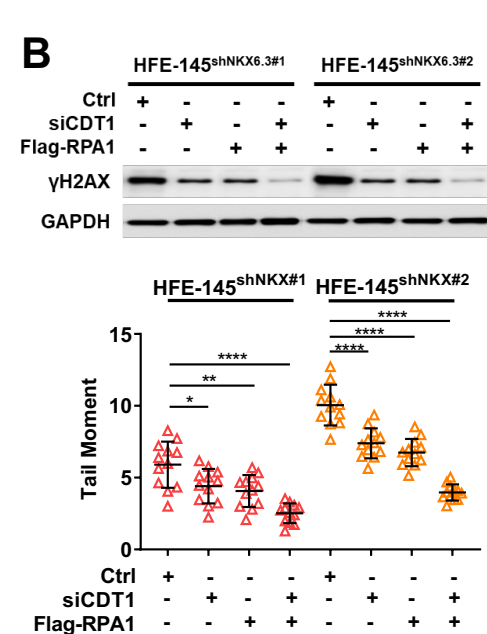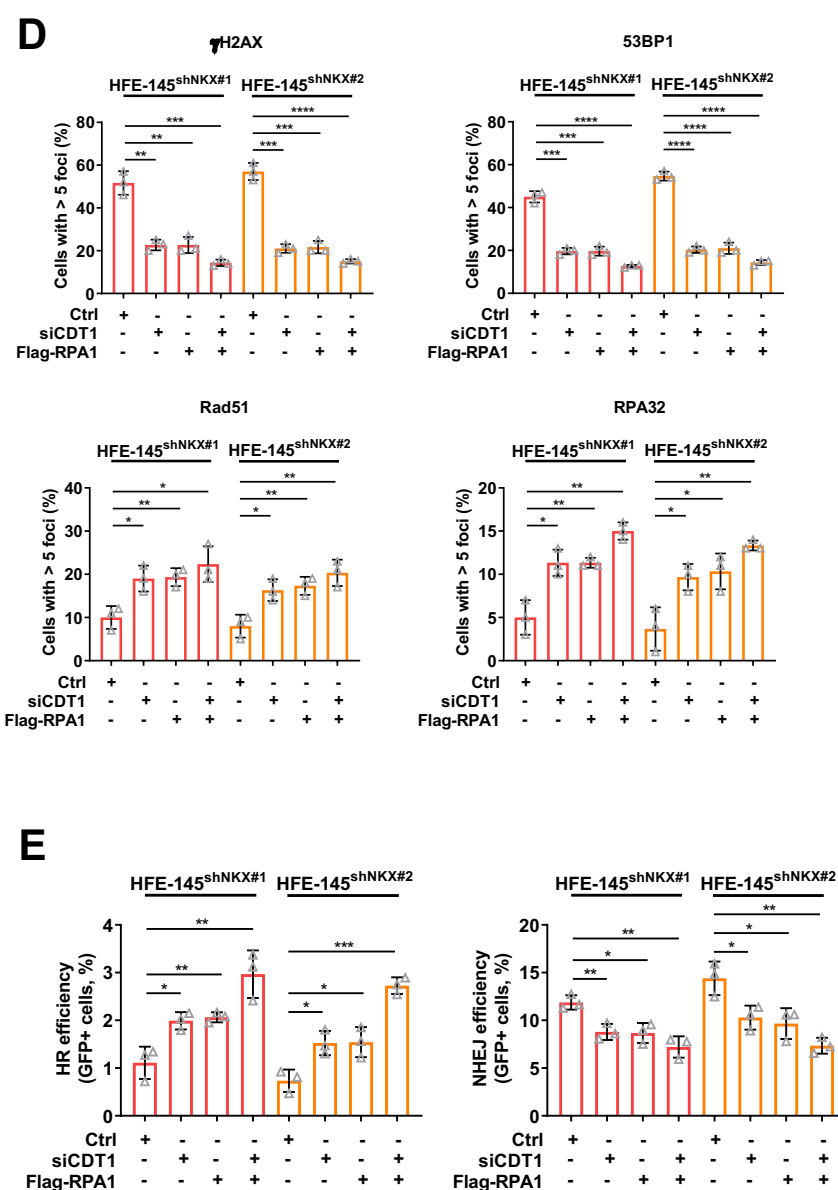

Supplement: Supplementary file 4 — Figure S3 [file 41389_2021_365_MOESM4_ESM.pdf]

NKX6.3

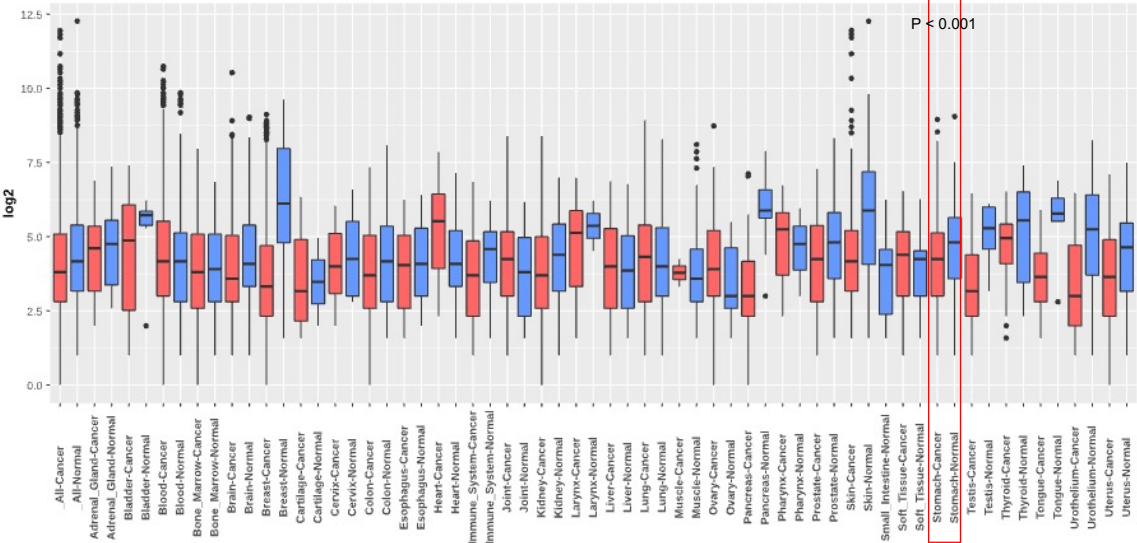

RPA1

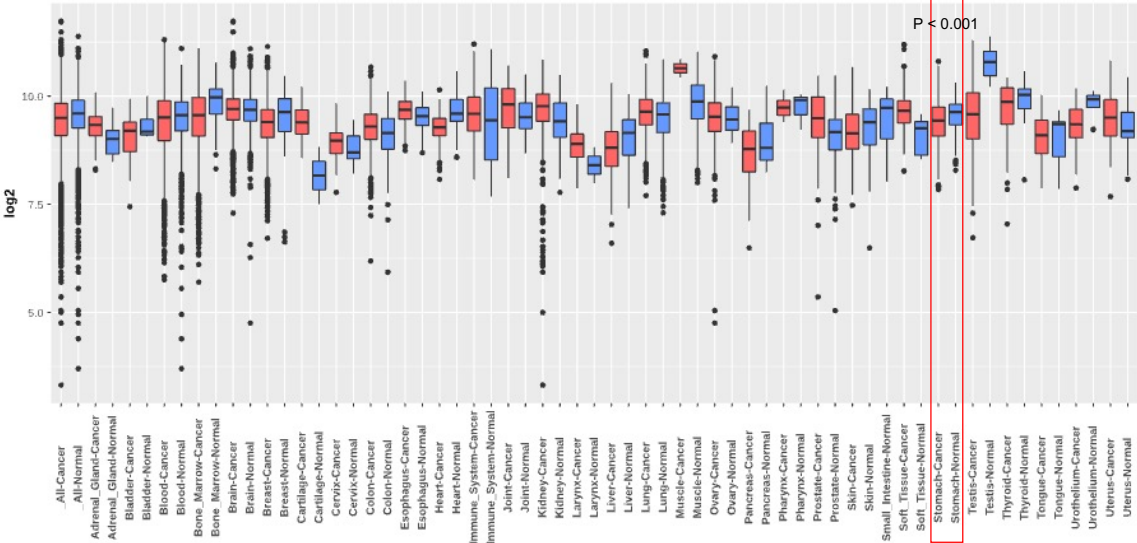

CDT1

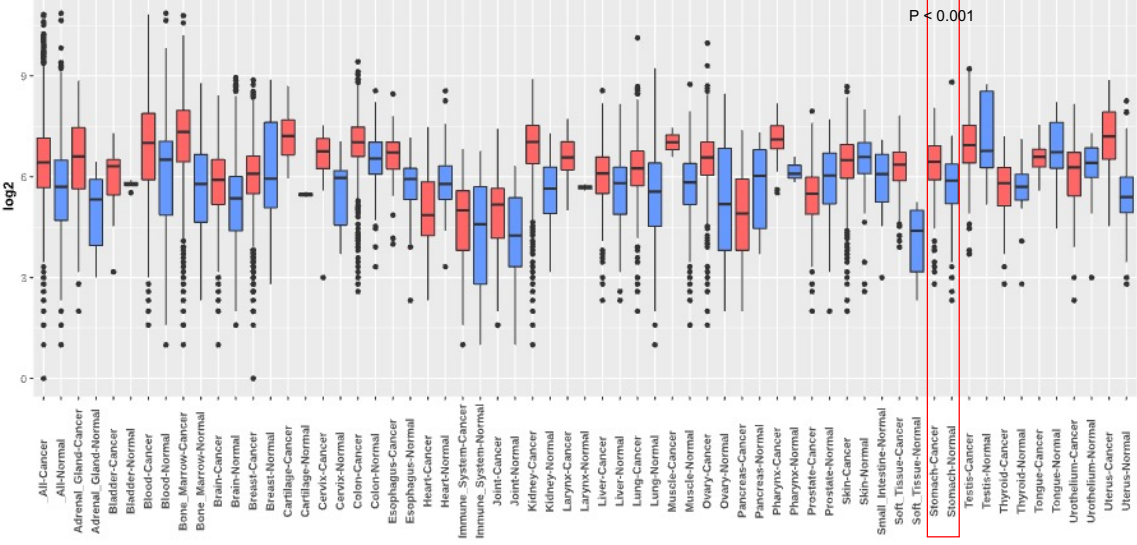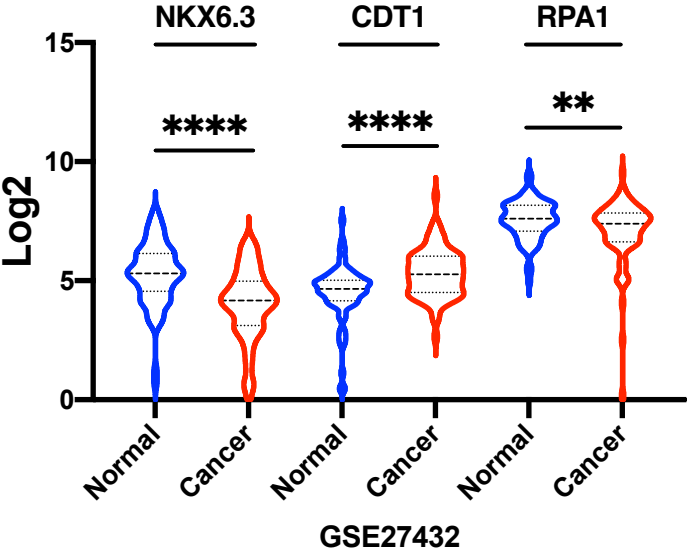

Supplement: Supplementary file 5 — Figure S4 [file 41389_2021_365_MOESM5_ESM.pdf]

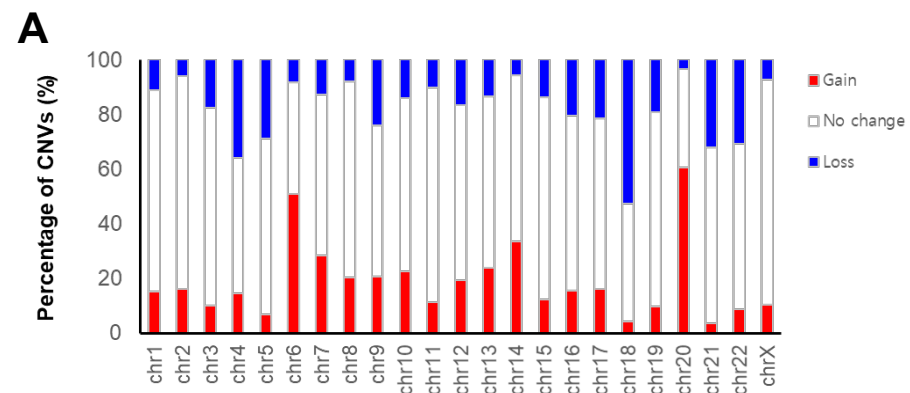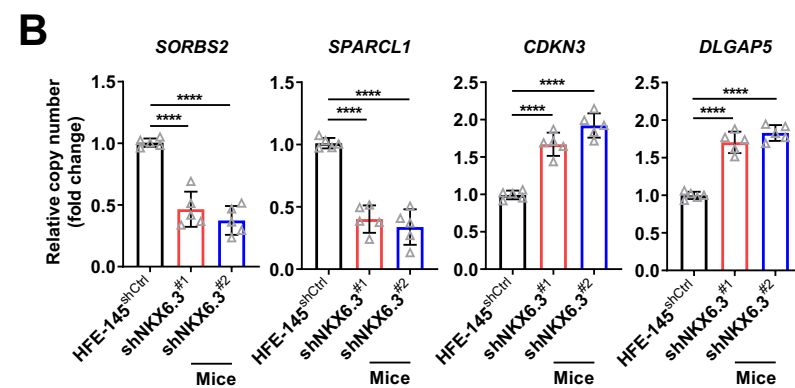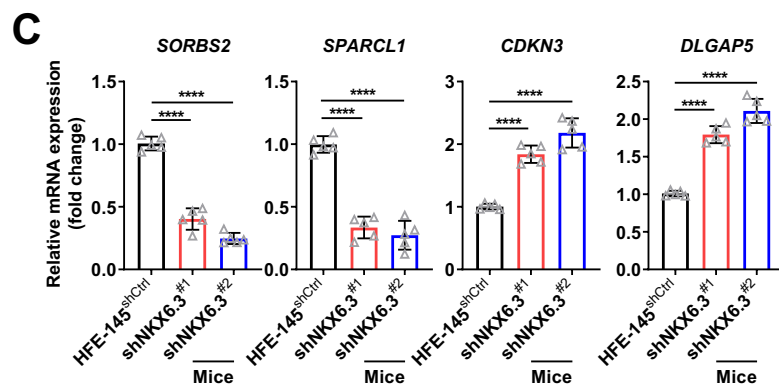

**D**

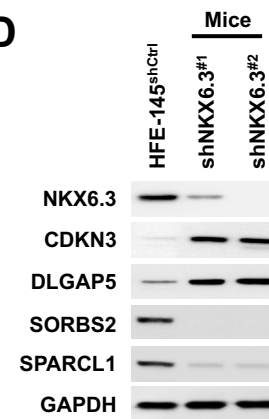

Supplement: Supplementary file 6 — Figure S5 [file 41389_2021_365_MOESM6_ESM.pdf]
